# Function Analysis of the *PR55*/*B* Gene Related to Self-Incompatibility in Chinese Cabbage Using CRISPR/Cas9

**DOI:** 10.3390/ijms23095062

**Published:** 2022-05-03

**Authors:** Na-Ri Shin, Yun-Hee Shin, Han-Seul Kim, Young-Doo Park

**Affiliations:** Department of Horticultural Biotechnology, Kyung Hee University, Yongin-si 1732, Korea; xhtjd232@naver.com (N.-R.S.); yunhee94@naver.com (Y.-H.S.); me1onr@naver.com (H.-S.K.)

**Keywords:** *Brassica rapa*, self-incompatibility, *PR55/B* gene, CRISPR/Cas9

## Abstract

Chinese cabbage, a major crop in Korea, shows self-incompatibility (SI). SI is controlled by the type 2A serine/threonine protein phosphatases (PP2As). The *PP2A* gene is controlled by regulatory subunits that comprise a 36 kDa catalyst C subunit, a 65 kDa regulatory A subunit, and a variety of regulatory B subunits (50–70 kDa). Among them, the PP2A 55 kDa B regulatory subunit (*PR55/B*) gene located in the A05 chromosome has 13 exons spanning 2.9 kb, and two homologous genes, *Bra018924* and *Bra014296*, were found to be present on the A06 and A08 chromosome, respectively. In this study, we performed a functional analysis of the *PR55*/*B* gene using clustered regularly interspaced short palindromic repeats/CRISPR-associated system 9 (CRISPR/Cas9)-mediated gene mutagenesis. CRISPR/Cas9 technology can be used to easily introduce mutations in the target gene. Tentative gene-edited lines were generated by the *Agrobacterium*-mediated transfer and were selected by PCR and Southern hybridization analysis. Furthermore, pods were confirmed to be formed in flower pollination (FP) as well as bud pollination (BP) in some gene-edited lines. Seed fertility of gene-edited lines indicated that the *PR55/B* gene plays a key role in SI. Finally, self-compatible T-DNA-free T_2_ gene-edited plants and edited sequences of target genes were secured. The self-compatible Chinese cabbage developed in this study is expected to contribute to Chinese cabbage breeding.

## 1. Introduction

Self-incompatibility (SI) is the most important system for preventing inbreeding in many flowering plants. Representative SI plants include the *Brassica* genus, which includes cabbage, radish, and Chinese cabbage. As the mechanism of SI has been utilized in F_1_ hybrid breeding, many studies have been conducted on the relevant practical aspects. Many relevant types of research have also been conducted on pollen stigma interactions, cell-cell interactions, and signal transduction [1,2,3]. Two closely linked polymorphic genes, the S-locus glycoprotein gene (*SLG*) and the S receptor kinase gene (*SRK*), located at the S-locus, function together, resulting in an SI response. The phosphorylation stage of *SRK* leading to the SI response depends on the activity of both the kinase and protein phosphatase (*PP*). Thus, inhibition of *PP* increases the phosphorylation level of *SRK* substrates and inactivates arm repeat-containing 1 (*ARC1*), resulting in self-compatibility [4,5].

Reversible protein phosphorylation, an essential regulatory mechanism in many cellular processes, can modify the properties of key regulatory proteins in specific pathways. In addition to protein kinases, PP is a highly regulated enzyme that plays an equally important role in controlling protein phosphorylation [6]. PP is grouped by substrate specificity as serine/threonine (Ser/Thr), tyrosine, and dual-specificity classes. The Ser/Thr-specific PPs are divided into four groups in eukaryotes: type 1 PP, type 2A PP (PP2A), type 2B PP (PP2B), and type 2C [7].

Functional genomics is an important research area that identifies the process by which genes are related to the phenotypes of organisms and provides biological and industrial value to determine gene availability [8,9]. Research directions for the functional analysis of genes include expression profiling, forward genetics, and reverse genetics. Expression profiling is the analysis of gene function through mRNA and protein expression levels. Forward genetics is the discovery of mutations in the genome and the identification of mutations associated with the target phenotype [10,11]. Reverse genetics mutates target genes to confirm the results. A line of mutant plants containing mutated copies of certain genes is then developed to confirm the gene function through the systematic suspension of target gene expression. These methods are particularly useful in analyzing genes whose functions are unclear because of the redundancy of functions among genes closely related to a single phenotype [12,13].

The development of knockdown mutations using T-DNA tagging or RNA interference (RNA*i*) is mainly used in reverse genetics, which reduces the transcription level of endogenous genes to analyze their function [14,15]. However, alternative technologies are needed because of the associated nonspecific insertion and unstable inheritance of T-DNA.

Clustered regularly interspaced short palindromic repeats/CRISPR-associated system 9 (CRISPR/Cas9) technology can be used to easily knock out the function of a specific gene because it does not have a complicated protein structure. The CRISPR/Cas9 system involves a single guide RNA (sgRNA), which includes a 20-nt sequence with a protospacer adjacent motif (PAM) and Cas9 protein. The sgRNA specifically binds the desired sequence, and Cas9 nuclease cuts 3 nucleotides upstream of the guide sequence [16,17,18].

Further, T-DNA can be isolated by generation advancement. Plasmids containing the CRISPR/Cas9 system have been successfully introduced into the mammalian genome [19,20]; the CRISPR/Cas9 system has also been applied to zebrafish [21] and *Caenorhabditis elegans* [22]. The CRISPR/Cas9 system has been reported to introduce sequence changes in several plants [23,24], including the model plants *Nicotiana benthamiana* [25] and *Arabidopsis thaliana* [26].

Chinese cabbage (*Brassica rapa* ssp. *pekinensis*) has been cultivated for a long time as an important vegetable crop in many Asian countries. Functional inhibition of SI is essential for breeding Chinese cabbage, which is a high-demand crop in Korea. SI is a phenomenon in which self-pollination cannot occur even though each reproductive organ is normal, and this is a common trait in *Brassica* crops. Currently, carbon dioxide or sodium chloride treatment [27,28] is performed to overcome SI, but new methods are needed considering the associated practicality, personal expenses, and environmental problems. Therefore, in this study, we examined the knockout of the PP2A 55 kDa B regulatory subunit (*PR55*/*B*) gene related to SI using gene-editing technology. The CRISPR/Cas9 system targeted the *PR55*/*B* gene related to SI. Molecular biological analysis was performed to confirm the change in the targeted sequence. Consequently, the CRISPR/Cas9 system functioned normally, and self-compatible Chinese cabbage was identified.

In this study, we confirmed that the PP2A 55 kDa B regulatory subunit, *PR55/B* gene, is closely related to the SI of Chinese cabbage using the CRISPR/Cas9 technique.

## 2. Results

### 2.1. Vector Construction and Agrobacterium-Mediated Transformation of Chinese Cabbage

**Figure 1:**
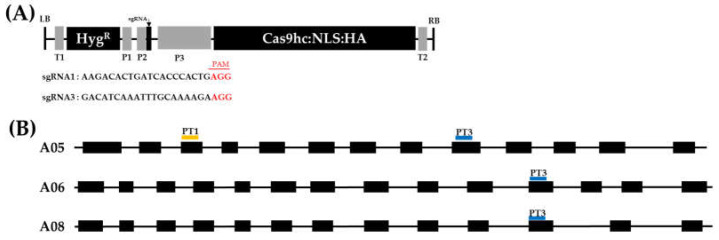
Vector structure and position of sgRNAs. (**A**) Detailed schematic representation of T-DNA. LB, left border; Hyg^R^, hygromycin resistance gene; Cas9hc:NLS:HA, human-codon-optimized Cas9 with the nuclear localization signal and an HA epitope; T1, NOS terminator; T2, 35S terminator. RB, right border. Hyg^R^, sgRNA, and Cas9hc:NLS:HA are under the control of NOS promoter (P1), Arabidopsis U6 promoter (P2), and 35S promoter (P3), respectively. (**B**) Summary of the genomic structure of the *PR55/B* gene and two homologous genes. Black box, exon regions; black line, intron regions; yellow bar, target site of sgRNA1; blue bar, target site of sgRNA3.

The sgRNA cassettes were designed, based on the ‘CT001′ genome sequence, to construct the gene-editing vector (Figure 1A). Analysis of the *PR55*/*B* gene (*Bra030425*) located on the A05 chromosome of ‘CT001′ was identified, and two homologous genes, *Bra018924* and *Bra014296*, were found to be present on the A06 and A08 chromosome, respectively. 

sgRNA1 (5′-AAG ACA CTG ATC ACC CAC TG-3′) was designed to target the *PR55*/*B* gene in the A05 chromosome, and sgRNA3 (5′-GAC ATC AAA TTT GCA AAA GA-3′) was designed to target the *PR55*/*B* gene and the two homologous genes, *Bra018924* in the A06 chromosome and *Bra014296* in the A08 chromosome (Figure 1B). Using the BioEdit program and FSTVAL, sgRNAs were confirmed to not target the exonic regions of other genes in ‘CT001′; thus, the probability of off-target effects was low. The T-DNA in the pHAtC vector, pPR55-Tra1 (PT1) and pPR55-Tra3 (PT3) with the designed sgRNA1 and sgRNA3 cassette, respectively, was inserted into inbred line ‘CT001′ by *Agrobacterium tumefaciens* mediated transformation.

### 2.2. Selection and Fertility Analysis of T_0_ Gene-Edited Chinese Cabbage Lines

**Figure 2:**
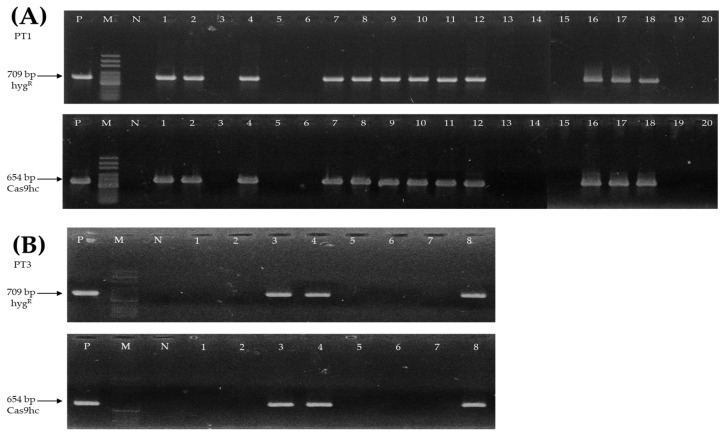
Selection of T_0_ gene-edited lines by PCR analysis. (**A**) PCR analysis with hyg^R^ and Cas9hc primer sets of T_0_ PT1 gene-edited lines. (**B**) PCR analysis with hyg^R^ and Cas9hc primer sets of T_0_ PT3 gene-edited lines. The 709 bp and 654 bp expected PCR products are indicated with an arrow, respectively. P, positive control; M, 100 bp DNA ladder; N, negative control; Numbering lane, tentative gene-edited lines.

**Figure 3:**
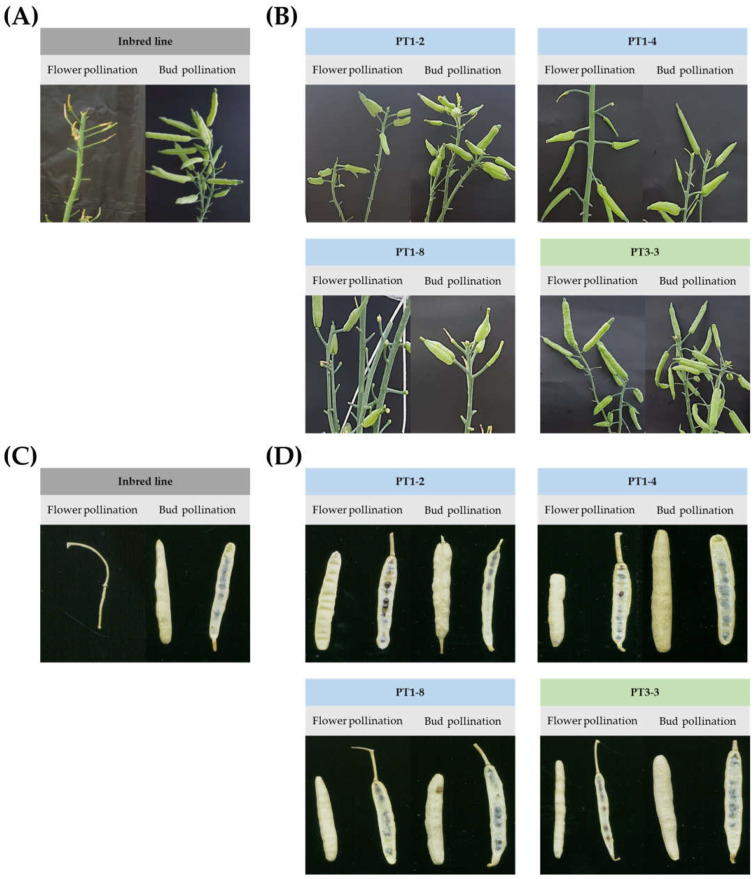
Self-compatibility analysis by FP and BP of inbred line ‘CT001′ and T_0_ gene-edited Chinese cabbage lines. (**A**) Pods of inbred line ‘CT001′ using FP and BP. (**B**) Pods of T_0_ gene-edited lines (PT1-2, -4, -8, and PT3-3) using FP and BP. (**C**) Seed formations of inbred line ‘CT001′ using FP and BP. (**D**) Seed formations of T_0_ gene-edited lines (PT1-2, -4, -8, and PT3-3) using FP and BP.

We obtained 20 and 8 tentative gene-edited lines for PT1 and PT3, respectively. PCR analysis was performed to select these lines. To confirm T-DNA insertion, two primer sets that could identify both hyg^R^ and Cas9hc:NLS:HA (Cas9hc) regions were used for PCR analysis. The 709 bp and 654 bp PCR amplicons were produced using the hyg^R^ and Cas9hc region primer sets. A total of 12 of 20 PT1 (Figure 2A) and 3 of the 8 PT3 (Figure 2B) gene-edited lines were identified.

To confirm the seed fertility of PT1 and PT3 gene-edited lines, flower pollination (FP) and bud pollination (BP) were conducted. While the inbred line, ‘CT001′, did not form pods upon FP in nature, the gene-edited lines showed the self-compatible phenotype when both BP and FP were carried out in this experiment. In ‘CT001′, pods were formed in BP, an SI-breaking method, but not in FP (Figure 3A,C). However, it was confirmed that gene-edited lines, particularly PT1-2, -4, -8, and PT3-3, formed pods in BP as well as in FP (Figure 3B,D). Furthermore, there was no significant difference in the seed setting between BP and FP. Analysis of the seed fertility in the inbred line ‘CT001′ and T_0_ gene-edited Chinese cabbage lines indicated that ‘CT001′ showed no seed fertility in FP and high fertility in the BP. In contrast, PT1-2, -4, -8, and PT3-3 lines showed higher seed fertility than ‘CT001′ in both BP and FP (Table 1). Based on the self-compatibility analysis, these lines were advanced to the T_1_ generation.

### 2.3. Confirmation of Sequence Change and Fertility Analysis in T_1_ Gene-Edited Chinese Cabbage Lines

The selection of T_1_ gene-edited lines was performed using the same method as the selection for T_0_ gene-edited lines (Figure S1 in Supplementary Materials). Some of the selected T_1_ gene-edited lines were analyzed for the target gene mutation, and self-compatibility was confirmed.

**Figure 4:**
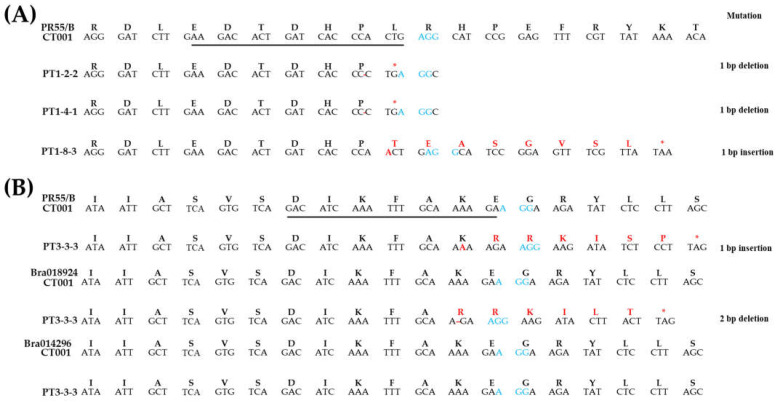
Analysis of mutation patterns in T_1_ gene-edited lines. (**A**) Confirmation of each nucleic and amino acid sequence of the *PR55*/*B* gene in PT1-2-2, -4-1, and -8-3. (**B**) Confirmation of each nucleic and amino acid sequence of the *PR55*/*B* gene, *Bra018924*, and *Bra014296* in PT3-3-3, respectively. The underline indicates sgRNA, and the blue font indicates the PAM sequence; the red font represents the presence of indel mutations and the resulting change of amino acid sequence.

PCR and RT-PCR amplicons were analyzed by the Sanger sequencing method. As a result, a single nucleotide deletion (PT1-2-2 and PT1-4-1) and insertion (PT1-8-3) were found in PT1. The deletion mutation introduces a stop codon, causing nonsense mutations. In addition, the insertion mutation changed leucine to threonine, resulted in a frameshift, and formed a stop codon in PT1-8 (Figure 4A). In PT3-3-3, no mutation was found in *Bra014296*, but a mutation was observed in both the *PR55/B* gene and *Bra018924* (Figure 4B). This mutation changed the amino acid sequence. A stop codon appeared in both the *PR55/B* gene and *Bra018924* following the indel mutations. Such an unexpected frameshift can interfere with the gene function.

**Figure 5:**
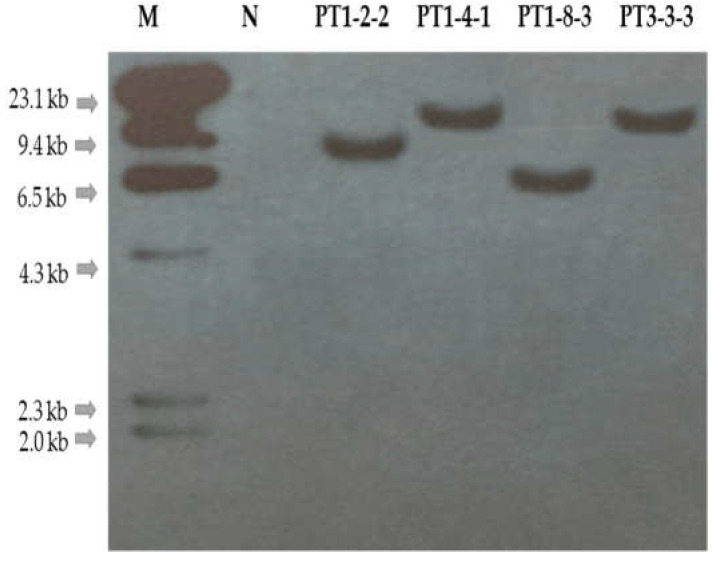
Southern hybridization analysis for identifying the copy number of T-DNA in the T_1_ gene-edited lines genome. A total of 30 μg genomic DNA was digested with *Eco*RI, then separated on a 1.0% agarose gel and blotted onto a Hybond N^+^ nylon membrane for hybridization with a probed [^32^P]-labeled 709 bp of hyg^R^ from a gene-editing vector. An approximate DNA molecular size marker is indicated on the left. M, λ *Hin*dIII molecular ladder; N, negative control; Lane, T_1_ gene-edited lines showing self-compatible phenotype and sequence mutations.

Southern hybridization analysis was performed to determine the T-DNA copy number of T_1_ gene-edited lines using a probe targeting the *hpt* gene of 709 bp; a non-transgenic gene-edited line was used as a negative control. In this analysis, no signal was detected in the negative control line, whereas one signal of 6.3–9.4 kb was clearly detected in PT1-2-2, -4-1, -8-3, and PT3-3-3 (Figure 5). Therefore, we concluded that the selected T_1_ gene-edited lines with one copy of T-DNA have a low probability of affecting other genes.

**Figure 6:**
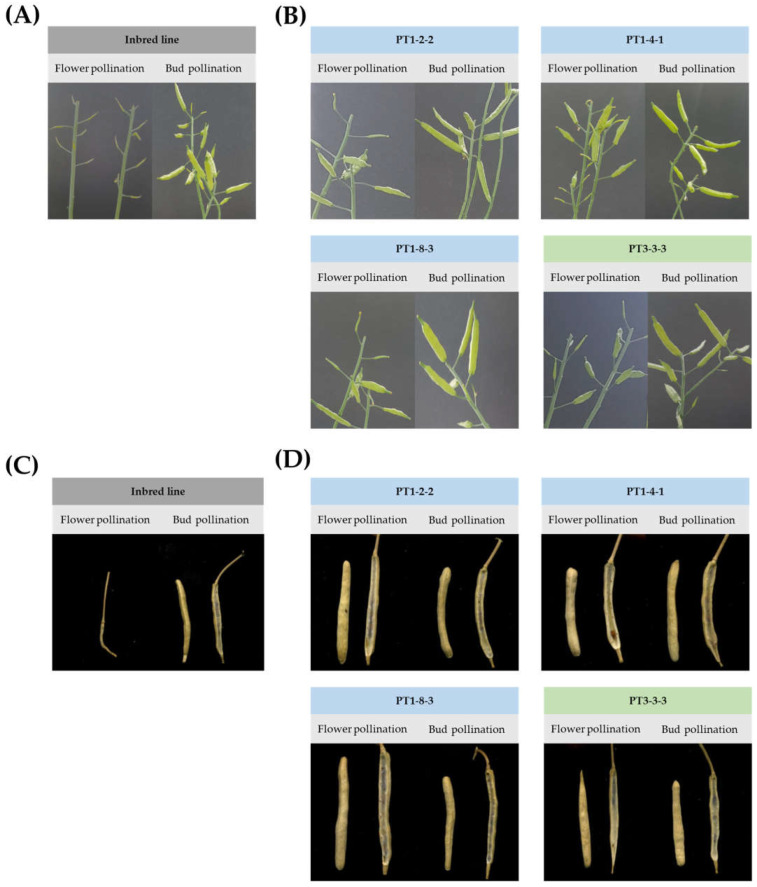
Self-compatibility analysis by FP and BP of T_1_ gene-edited Chinese cabbage lines. (**A**) Pods of inbred line ‘CT001′ using FP and BP. (**B**) Pods of T_1_ gene-edited lines (PT1-2-2, -4-1, -8-3, and PT3-3-3) using FP and BP. (**C**) Seed formations of inbred line ‘CT001′ using FP and BP. (**D**) Seed formations of T_1_ gene-edited lines (PT1-2-2, -4-1, -8-3, and PT3-3-3) using FP and BP.

After confirmation of sequence changes and T-DNA insertion, the selected T_1_ gene-edited lines were subjected to cold treatment for 2 months. FP and BP were conducted using the same method as for T_0_ gene-edited lines. When both FP and BP were conducted on ‘CT001′, pods were formed in BP (Table 1), an SI-breaking method, but none were formed in FP (Figure 6A,C). However, the T_1_ generation, especially PT1-2-2, -4-1, -8-3, and PT3-3-3 lines, showed a good formation of pods with both BP and FP (Figure 6B,D).

The seed fertility of the gene-edited lines was higher than that of inbred line ‘CT001′ (Table 2). When FP and BP were performed in the gene-edited lines, there was no significant difference in the shape or number of pods between FP and BP. There was also no external phenotypic difference in the gene-edited lines compared to the inbred line ‘CT001′. This phenomenon of T_1_ gene-edited lines was derived from the acquisition of self-compatibility caused by *PR55/B* gene inhibition. These results confirmed that using the CRISPR/Cas9 system with the *PR55/B* gene-editing vector allowed the development of self-compatible Chinese cabbage.

### 2.4. Inheritance of Sequence Change in T-DNA-Free T_2_ Gene-Edited Chinese Cabbage Lines

**Figure 7:**
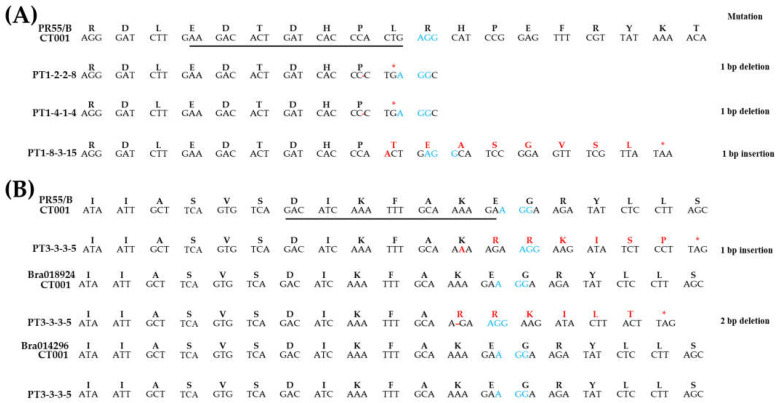
Analysis of mutation patterns in T_2_ gene-edited lines. (**A**) Confirmation of each nucleic and amino acid sequence of *PR55/B* gene, *Bra018924* and *Bra014296* in PT1-2-2-8, -4-1-4, and -8-3-15, respectively. (**B**) Confirmation of each nucleic and amino acid sequence of *PR55/B* gene, *Bra018924*, and *Bra014296* in PT3-3-3-5. The underline indicates sgRNA, and the blue font indicates the PAM sequence; the red font represents the presence of indel mutations and the resulting change of amino acid sequence.

Two primer sets targeting hyg^R^ and Cas9hc regions were used for the PCR analysis of T_2_ plants to select T-DNA-free self-compatible lines. The PCR amplicons showed that 8 (12.1%) of 66 T_2_ plants could not be amplified from the transferred pPR55-Tra1 construct (Figure S2A,B in Supplementary Materials). Likewise, no hyg^R^ amplicon was generated in 2 (9.1%) out of 22 T_2_ plants derived from the pPR55-Tra3 construct (Figure S2A,B in Supplementary Materials). As with the DNA sequence change identified at the target site of the T_1_ generation, the sequence change was confirmed to be the same in the T_2_ lines, but it was confirmed that there was no T-DNA (Figure 7). These T-DNA-free T_2_ gene-edited lines also showed self-compatibility, and it was confirmed that pods and seeds were normally formed in both BP and FP. These findings suggest that genetic segregation could be efficiently used to acquire T-DNA-free gene-edited plants during the generation process.

## 3. Discussion

From an evolutionary viewpoint, SI benefits evolution through cross-fertilization. Plants with other genetic material are crossed, increasing their chances of surviving in nature. However, this characteristic poses difficulties to humans during crop handling. When breeding inbred lines and F_1_ hybrids, it is necessary to overcome SI [29,30]. The methods currently used to break SI involve dealing with carbon dioxide, sodium chloride, or BP [27,28]. However, chemical methods can cause environmental issues; further, they are difficult to sustain and require alternative management. Thus, various studies of genes associated with the SI mechanism have been conducted to control SI [31,32,33,34,35]. In Chinese cabbage, studies have been conducted on the SI mechanisms, mainly with the *SLG* and *SRK* genes. To our knowledge, the present study is the first to target *PR55*/*B* genes using the CRISPR/Cas9 system. 

The PP2A protein can control cell metabolism and biological processes, such as the cell cycle, transcription, translation, and signal transduction [36,37]. The PP2A protein comprises a 36 kDa catalyst C subunit, a 65 kDa regulatory A subunit, and a variety of regulatory B subunits (50–70 kDa) [38,39,40] and is strictly controlled by regulatory subunits [39,41]. The PP2A protein is identified from *B*. *napus* seeds, preferentially dephosphorylates the α-subunit of phosphorylase kinase and is strongly inhibited by in vitro okadaic acid (OA) [42,43]. OA is a naturally occurring phosphatase inhibitor that can penetrate living cells and is the most widely used phosphatase inhibitor [44]. Treatment with OA completely overcomes SI, indicating that the PP2A protein is involved in the SI mechanism [45].

The PP2A protein is associated with the interaction between pollen and pistil during pollination. The SI reaction appears as the activation signal of the SRK kinase domain and is transmitted to the stigma, where it interacts between SRK and ARC1. The SRK activation occurs by the dephosphorylation of SRK and phosphorylation of ARC1 by PP [4,5,46]. Therefore, among various types of PPs, loss of function of the PP2A protein negatively affects the SRK and ARC1 activation. In gene-edited Chinese cabbage lines, SI was thought to be triggered by inactivated ARC1. Phenotype analysis related to the SI caused by the *PP2A* gene was performed at the BP and FP stages in the *PR55/B* gene knock-out and wild type [46]. The *PR55/B* gene knock-out line-producing seeds could be regarded as the loss of SI due to inhibition of the *PP2A* gene and inactivation of *ARC1,* suggesting that the *PR55/B* gene was closely related to the SI in Chinese cabbage.

In this study, the *PR55/B* gene located in the A05 chromosome has 13 exons spanning 2.9 kb, and the homologous genes of the *PR55/B* gene were identified. *Bra018924* located in the A06 chromosome has 14 exons, and *Bra014296* located in the A08 chromosome has 13 exons. Two gene-editing vectors were constructed with sgRNAs targeting different exons; sgRNA1 and sgRNA3 targeted the *PR55*/*B* gene and all three homologous genes, respectively. Random mutations were produced by non-homologous end-joining in T_1_ plants that exhibited self-compatible phenotypes. Most mutations that occurred were point mutations, including insertion, deletion, and transversion. The occurrence of an indel mutation in the *PR55/B* gene produced an early stop codon, resulting in the loss of function of the PR55/B protein. Further, the occurrence of frame-shift mutations in PT3 3-3 causes structural transitions and introduces an early stop codon in the PR55/B protein (Figure 4).

Similarly, the production of early stop codons in drought-responsive RING Protein 1 (*DRR1*) damages drought stress resistance by causing the accumulation of ubiquitous insoluble proteins in *Arabidopsis* [47]. The introduction of a premature stop codon in the open reading frame of *Solanum lycopersicum* SRFR1 (*SlSRFR1*) was reported to increase the expression of defense genes in the salicylic acid pathway and to enhance resistance to *Pseudomonas syringae* in tomato [48]. In addition, another study obtained the desired phenotype with prolonged basic vegetative growth periods at low latitudes in japonica rice through frameshift and frame deletion mutations of the early heading date 1 gene (*EHd1*) [49].

To increase the possibility of frameshift occurring through the generation of indels, multiple sites may be targeted using two or more sgRNAs [50,51,52]. Targeting multiple sites has been suggested to improve the genome editing efficiency in various crops such as maize [53], grape [54], and cabbage [55]. Further, targeting gene families with similar functions increases the efficiency of gene editing. Multiple gene editing was performed efficiently in the KARRIKIN INSENSITIVE 2 LIKE (*PpKAI2L*) gene family, which encodes the receptors and candidate receptors of butenolide compounds such as strigolactones or karrikins [56]. However, some previous reports have suggested that targeting multiple genes with a single sgRNA can reduce the genome editing efficiency because of a variety of reasons [57,58]. Similarly, sgRNA3, which targets three genes, was found to have worked normally for only two genes, *PR55/B* and *Bra018924*, in this study. However, it is possible that the indels caused by a single sgRNA produced a frameshift of the target gene, resulting in phenotypic defects. The editing of multiple sites using a single sgRNA has also been reported in poplar [59]. In this study, we used the CRISPR/Cas9 system to successfully target multiple sites of the *PR55*/*B* gene in *Brassica rapa* using a single sgRNA. Indel mutations, which lead to a frameshift, were detected in the *PR55/B* gene and *Bra018924* (Figure 4B). Thus, we consider the CRISPR/Cas9 system to be effective because it can edit target gene sequences and can be applied for the sequence editing of gene families.

Using PCR analysis, we confirmed that many gene-edited plants still had T-DNA (Figure S1 in Supplementary Materials). Because T-DNA insertion occurs randomly for the number of T-DNA copies and insertion locations, unexpected genetic mutations are possible [52,60,61]. It is easier to analyze the insertion location when a single copy of T-DNA is inserted compared to when multiple copies of T-DNA are inserted [61,62]; furthermore, we were able to isolate T-DNA fragments from the plant genome. T-DNA-free genome-edited plants can be produced through generational advancement. T-DNA-separated plants are more likely to be free from GMO issues and can be evaluated positively by the seed industry.

Genetic analysis of ‘CT001′ identified each off-targeting region targeted by sgRNA, and the off-targeting probability was also very low in the program; therefore, it would not have targeted other genes. Further, when CRISPR/Cas9 is applied to plants, the off-targeting probability is very low, and many studies have demonstrated the precision of CRISPR/Cas9 [63,64]. Regardless of the pollination method, the T_0_ and T_1_ generations of the gene-edited lines formed pods and seeds almost normally. Therefore, we concluded that the region where the CRISPR/Cas9 vector was destroyed was stably maintained and that SI was broken. The results of this study also confirmed that CRISPR/Cas9 is effective in gene function analysis and can be applied to breed useful Chinese cabbage and other useful crops as well.

Stable insertion and inheritance of T-DNA into plant genomes are essential for useful traits. In addition, the downregulation of genes based on principles such as RNA interference is caused by T-DNA insertion. In particular, a self-compatible phenotype has been introduced using the RNA*i* technique to regulate the *PR55*/*B* gene in Chinese cabbage [46]. This technique is an effective way to control the gene expression level but cannot achieve the genetic stability of GM crops owing to the insertion of T-DNA into the plant genome [65]. However, with simple principles and excellent precision, the CRISPR/Cas9 method enables T-DNA-free gene modification, which is not possible with conventional genetic engineering. T-DNA, including the sgRNA cassette, can be easily removed from the plant genome by separation in the next generation after gene-editing events induced by CRISPR/Cas9. Gene editing, especially the CRISPR/Cas9 system, is an innovative technology for the development of non-GM crops and has been used in all types of crops, including vegetables, fruit trees, and flowers [26,58,66]. The CRISPR/Cas9 system is a useful technique for modifying various characteristics in plants. There are studies on T-DNA-free gene mutations in various traits, such as inducing early flowering [67,68] or acquiring disease resistance [69,70,71] using the CRISPR/Cas9 system. Thus, T-DNA-free plants produced using the CRISPR/Cas9 system can mitigate biological safety issues and facilitate regulatory approval for commercial applications.

## 4. Materials and Methods

### 4.1. Target Site Selection and Construction of PR55/B Gene-Editing Vectors

To regulate the SI of Chinese cabbage (*Brassica rapa* ssp. *pekinensis*), the *PR55/B* gene was selected as the target gene. To design sgRNAs targeting the *PR55/B* gene, the number of *PR55/B* gene copies in the ‘CT001′ genome was confirmed using FSTVAL (http://bioinfo.mju.ac.kr/fstval/ accessed on 2 May 2018). The homology of each copy and the *PR55/B* gene were analyzed using the Brassica database (http://brassicadb.cn/#/ accessed on 2 May 2018). The exon and intron regions of each identified homologous gene were analyzed to design sgRNA with target regions affecting the gene expression using Softberry (http://www.softberry.com/ accessed on 10 May 2018). Further, candidate sgRNAs in exons containing PAM sequences were designed using CRISPR direct (http://crispr.dbcls.jp/ accessed on 11 May 2018). The off-targeting probability was analyzed using FSTVAL (http://bioinfo.mju.ac.kr/fstval/ accessed on 16 May 2018) and the BioEdit program based on the pseudomolecules of ‘CT001′ to ensure that sgRNAs could not affect the expression of other genes.

sgRNA1 was designed to target the third exon of the *B*. *rapa PR55/B* gene, and sgRNA3 was designed to target all three homologous genes of the *PR55/B* gene to break out of SI. The sgRNA cassette comprising the AtU6 promoter and sgRNA with the scaffold was cloned into the pHAtC plasmid vector [72] (Figure 1A). The designed 369 bp sgRNA cassettes were synthesized by Macrogen Co. (Seoul, Korea) and cloned into the *Sac*I site of the binary vector. The hygromycin phosphotransferase (*hpt*) gene was inserted as a selection marker to select gene-edited lines. The modified freeze-thaw method [73] was used to transfer the constructed gene-editing vectors into *Agrobacterium tumefaciens* LBA4404.

### 4.2. PCR and RT-PCR Analysis of Gene-Edited Chinese Cabbage Lines

The inbred line ‘CT001′ of Chinese cabbage was used to develop gene-edited Chinese cabbage lines. The seeds of ‘CT001′ were sterilized with 70% EtOH and 30% sodium hypochlorite solution and then sown in an MS basal medium [74]. Chinese cabbage transformation was conducted using effective methods [9,75]. The tentative gene-edited plants were grown in a greenhouse at Kyung Hee University (Yongin, Korea). Genomic DNA (gDNA) was extracted from the leaves of inbred line ‘CT001′ and from the tentative gene-edited plants using a modified method [76]. Two primer sets targeting the hyg^R,^ and Cas9hc regions of the gene-editing vector were used for the PCR analysis (Table S1 in Supplementary Materials). For PCR analysis, gDNA and specific primer sets were used with the Maxime PCR PreMix Kit (iNtRON Biotechnology, Seongnam, Korea). The PCR conditions were as follows: initial denaturation at 95 °C for 10 min, 35 cycles of 95 °C for 30 s, 62 °C for 1 min, 72 °C for 40 s, and a final extension step at 72 °C for 5 min. The PCR products were analyzed by electrophoresis with 1% agarose gel with ethidium bromide staining.

The total RNA was extracted from the leaves of ‘CT001′ and the gene-edited lines using the TaKaRa MiniBEST Plant RNA Extraction kit (Takara, Otsu, Japan), according to the manufacturer’s instructions. The RNA purity and concentration were measured using a Nanodrop ND-1000 spectrophotometer (NanoDrop Technologies, Wilmington, SA, Australia). The extracted RNA was used to synthesize cDNA with the HiSenScript RH[-] RT PreMix Kit (iNtRON Biotechnology, Seongnam, Korea). The cDNA synthesis conditions were as follows: reverse transcription at 42 °C for 1 h and RNase inactivation extension at 85 °C for 10 min. Specific primer sets targeting each exon were used for RT-PCR analysis (Table S1 in Supplementary Materials). For RT-PCR analysis, cDNA and specific primer sets were used with the Maxime PCR PreMix Kit (iNtRON Biotechnology, Seongnam, Korea). RT-PCR conditions were as follows: initial denaturation at 95 °C for 10 min, 35 cycles of 95 °C for 30 s, 56 °C for 30 s, 72 °C for 20 s, and a final extension step at 72 °C for 5 min. Electrophoresis with 1% agarose gel and ethidium bromide staining was used to analyze the results.

### 4.3. Southern Hybridization Analysis for Identifying the Number of T-DNA Copies

To determine the T-DNA copy number in the genome of gene-edited Chinese cabbage lines by Southern hybridization analysis, 30 μg of genomic DNA were digested with 3 μL of *Hin*dIII overnight at 37 °C. The digested genomic DNA was separated by size on a 1% agarose gel with a lambda *Hin*dIII molecular marker to estimate the size accurately. After electrophoresis was carried out for 8 h at 30 V, the gel was depurinated, denatured, and neutralized. Denatured DNA was blotted onto a Hybond N^+^ nylon membrane (Amersham Pharmacia, Buckinghamshire, Little Chalfont, UK). The 709 bp fragment of hyg^R^ was used as a probe, which was labeled with ^32^P-dCTP using the BcaBEST Labelling kit (TaKaRa, Otsu, Japan). The hybridized membrane was washed in a shaking incubator at 60 °C. The washed nylon membrane was then exposed to an X-ray film at −80 °C for 72 h for autoradiography and then visualized.

### 4.4. Self-Compatibility Analysis of Gene-Edited Chinese Cabbage Lines

To examine the self-compatibility of gene-edited lines, plants were cultivated in a greenhouse at ‘Hankookseed’ Company (Pyeongtaek, Korea). The environmental conditions for pollination were the same as those in a previous study [46]. The selected gene-edited Chinese cabbage lines progressed to the T_1_ generation by FP and BP. BP was conducted by opening the bud before flowering and pollinating pollen from an open flower of the same individual line to the stigma of the opened bud. FP was carried out by transferring pollen from the anther to the stigma of the same flower after flowering. For each gene-edited line and control, seed fertility was calculated by dividing the number of seeds by the number of pods. using the following formula: seed fertility = number of seeds ÷ number of pods.

### 4.5. Sequence Change Analysis of Gene-Edited Chinese Cabbage Lines

To confirm the sequence change of target genes, DNAs and RNAs isolated from gene-edited lines were used for PCR and RT-PCR analysis, respectively. PCR and RT-PCR analyses were conducted with cSEQ primer sets (Table S1 in Supplementary Materials), and the amplicons were produced at expected sizes (Figure S1 in Supplementary Materials). The amplicons were eluted using the P&C Multiple Elution Kit (Biosolution, Suwon, Korea). The sequences were obtained from BTSeq (Celemics, Seoul, Korea) and analyzed. Through the alignment of nucleic and amino acid sequences, the sequences of ‘CT001’ and gene-edited lines were compared and analyzed.

## 5. Conclusions

In this study, using CRISPR/Cas9 targeting the *PR55/B* gene family, self-compatible gene-edited lines of Chinese cabbage were developed, and molecular analysis was conducted. The gene-editing vector was constructed, and T-DNA was inserted into Chinese cabbage through *Agrobacterium*-mediated transformation. Self-compatibility analysis was also performed using FP and BP in the gene-edited lines selected by the PCR analysis. Finally, it was confirmed that one copy of T-DNA was inserted into the plant genome, which introduced indel mutations in the target gene. In this study, indel mutation caused a stop codon or frameshift occurrence. The occurrence of an indel mutation in the *PR55/B* gene produced an early stop codon, resulting in the loss of function of the PR55/B protein. Furthermore, the occurrence of frameshift mutations in PT3-3-3 triggered structural transitions and introduced an early stop codon. T_1_ generation, especially the PT1-2-2, -4-1, -8-3, and the PT3-3-3 lines, showed no significant difference in the shape or number of pods between FP and BP. In addition, it was confirmed that the DNA sequence changes were the same in T_1_ and T_2_ plants, which were T-DNA free. Consequently, we confirmed that the *PR55/B* gene was an important factor in SI and that the CRISPR/Cas9 system worked stably in Chinese cabbage. Therefore, it was possible to develop self-compatible Chinese cabbage lines by targeting the *PR55/B* genes related to SI through the CRISPR/Cas9 system. The results of this study can be directly applied to the breeding program and will be helpful in the development of new breeding techniques. 

## 6. Patents

We are in the process of obtaining a patent for the data on the method of using a plant transformation vector for gene editing SI characteristics in Chinese cabbage and their applications in Korea (patent number 10-2019-0127450).

## Figures and Tables

**Table 1:** Seed fertilities of inbred line ‘CT001′ and T_0_ gene-edited Chinese cabbage lines.

Line	Methods of Pollination ^z^	Pods	Seeds	Seed Fertility ^y^
IB	FP	0	0	0
	BP	49	121	2.47
IB	FP	0	0	0
	BP	32	72	2.25
PT1-2	FP	32	79	2.47
	BP	24	67	2.80
PT1-4	FP	48	76	1.58
	BP	32	50	1.56
PT1-8	FP	26	64	2.46
	BP	40	105	2.63
PT3-3	FP	40	72	1.80
	BP	24	50	2.08

^z^: FP, flower pollination; BP, bud pollination. ^y^: Seed fertility, the total number of seeds/total number of pods.

**Table 2:** Seed fertilities of inbred line ‘CT001′ and T_1_ gene-edited Chinese cabbage lines.

Line	Methods of Pollination ^z^	Pods	Seeds	Seed Fertility ^y^
IB	FP	0	0	0
	BP	52	108	2.08
IB	FP	0	0	0
	BP	30	55	1.83
PT1-2-2	FP	30	52	1.73
	BP	28	49	1.75
PT1-4-1	FP	43	70	1.63
	BP	49	90	1.84
PT1-8-3	FP	30	55	1.83
	BP	58	115	1.98
PT3-3-3	FP	40	72	1.80
	BP	38	70	1.84

^z^: FP, flower pollination; BP, bud pollination. ^y^: Seed fertility, the total number of seeds/total number of pods.

## Data Availability

The constructed gene-editing vectors generated during the current study were deposited in the National Agricultural Biotechnology Information Center (NABIC) (http://nabic.rda.go.kr) in Rural Development Administration (RDA), Korea, with the accession numbers, pPR55-Tra1:NU-1244 and pPR55-Tra3:NU-1304. The nucleotide information is publicly available.

## Supplementary Materials

The following are available online at https://www.mdpi.com/article/10.3390/ijms23095062/s1.

## References

[1] Takayama S., Isogai A. (2005). Self-incompatibility in plants. Annu. Rev. Plant Biol..

[2] Hiscock S.J., Allen A.M. (2008). Diverse cell signalling pathways regulate pollen-stigma interactions: The search for consensus. New Phytol..

[3] Igic B., Lande R., Kohn J.R. (2008). Loss of self-incompatibility and its evolutionary consequences. Int. J. Plant Sci..

[4] Vanoosthuyse V., Tichtinsky G., Dumas C., Gaude T., Cock J.M. (2003). Interaction of calmodulin, a sorting nexin and kinase-associated protein phosphatase with the *Brassica oleracea S* locus receptor kinase. Plant Physiol..

[5] Sehgal N., Singh S. (2018). Progress on deciphering the molecular aspects of cell-to-cell communication in *Brassica* self-incompatibility response. 3 Biotech.

[6] Janssens V., Goris J. (2001). Protein phosphatase 2A: A highly regulated family of serine/threonine phosphatases implicated in cell growth and signalling. Biochem. J..

[7] Rundle S.J., Nasrallah M.E., Nasrallah J.B. (1993). Effects of inhibitors of protein serine/threonine phosphatases on pollination in *Brassica*. Plant Physiol..

[8] Ku J.J., Park Y.H., Park Y.D. (2006). A non-antibiotic selection system uses the phosphomannose-isomerase (PMI) gene for *Agrobacterium*-mediated transformation of Chinese cabbage. J. Plant Biol..

[9] Yu J.G., Lee G.H., Kim J.S., Shim E.J., Park Y.D. (2010). An insertional mutagenesis system for analyzing the Chinese cabbage genome using *Agrobacterium* T-DNA. Mol. Cells..

[10] Singh V.K., Khan A.W., Saxena R.K., Sinha P., Kale S.M., Parupalli S., Kumar V., Chitikineni A., Vechalapu S., Kumar C.V.S. (2017). Indel-seq: A fast-forward genetics approach for identification of trait-associated putative candidate genomic regions and its application in pigeonpea (*Cajanus cajan*). Plant Biotechnol. J..

[11] Gajewska P., Janiak A., Kwasniewski M., Kędziorski P., Szarejko I. (2018). Forward genetics approach reveals a mutation in bHLH transcription factor-encoding gene as the best candidate for the root hairless phenotype in barley. Front. Plant Sci..

[12] Gilchrist E., Haughn G. (2010). Reverse genetics techniques: Engineering loss and gain of gene function in plants. Brief. Funct. Genom..

[13] Lloyd J., Meinke D. (2012). A comprehensive dataset of genes with a loss-of-function mutant phenotype in Arabidopsis. Plant Physiol..

[14] An S., Park S., Jeong D.H., Lee D.Y., Kang H.G., Yu J.H., Hur J.H., Kim S.R., Kim Y.H., Lee M.O. (2003). Generation and analysis of end sequence database for T-DNA tagging lines in rice. Plant Physiol..

[15] Yu J.G., Lee G.H., Park Y.D. (2012). Comparison of RNA interference-mediated gene silencing and T-DNA integration techniques for gene function analysis in Chinese cabbage. Hortic. Sci. Technol..

[16] Jinek M., Chylinski K., Ronfara I., Hauer M., Doudna J.A., Charpentier E.A. (2012). programmable dual-RNA-guided DNA endonuclease in adaptive bacterial immunity. Science.

[17] Jinek M., Jiang F., Taylor D.W., Sternberg S.H., Kaya E., Ma E., Anders C., Hauer M., Zhou K., Lin S. (2014). Structures of Cas9 endonucleases reveal RNA-mediated conformational activation. Science.

[18] Nishimasu H., Ran F.A., Hsu P.D., Konermann S., Shelata S.I., Dohmae N., Ishitani R., Zhang F., Nureki O. (2014). Crystal structure of Cas9 in complex with guide RNA and target DNA. Cell.

[19] Cong L., Ran F.A., Cox D., Lin S., Barretto R., Habib N., Hsu P.D., Wu X., Jiang W., Marraffini L.A. (2013). Multiplex genome engineering using CRISPR/Cas systems. Science.

[20] Mali P., Yang L., Esvelt K.M., Aach J., Guell M., DiCarlo J.E., Julie E.N., Church G.M. (2013). RNA-guided human genome engineering via Cas9. Science.

[21] Hrusha A., Krawitz P., Rechenberg A., Heinrich V., Hecht J., Haass C., Schmid B. (2013). Efficient CRISPR/Cas9 genome editing with low off-target effects in zebrafish. Development.

[22] Friedland A.E., Tzur Y.B., Esvelt K.M., Colaiacovo M.P., Church G.M., Calarco J.A. (2013). Heritable genome editing in *C.*
*elegans* via a CRISPR-Cas9 system. Nat. Methods.

[23] Zhou H., He M., Li J., Chen L., Huang Z., Zheng S., Zhu L., Ni E., Jiang D., Zhao B. (2016). Development of commercial thermos-sensitive genic male sterile rice accelerates hybrid rice breeding using the CRISPR/Cas9-mediated TMS5 editing system. Sci. Rep..

[24] Qin X., Li W., Liu Y., Tan M., Ganal M., Chetelat R.T. (2018). A farnesyl pyrophosphate synthase gene expressed in pollen functions in S-RNase-independent unilateral incompatibility. Plant J..

[25] Nekrasov V., Staskawicz B., Weigel D., Jones J.D., Kamoun S. (2013). Targeted mutagenesis in the model plant *Nicotiana benthamiana* using Cas9 RNA-guided endonuclease. Nat. Biotechnol..

[26] Mao Y., Zhang H., Xu N., Zhang B., Gou F., Zhu J.K. (2013). Application of the CRISPR-Cas system for efficient genome engineering in plants. Mol. Plant.

[27] Kim Y.G., Kim D.H., Park C.G., Yeo J.H., Ahn Y.S., Park H.K. (2009). Effect on Breaking of Self-Incompatibility by Old-Flower Pollination, NaCl and *CO_2_* Treatment in *Astragalus membranaceus* Bunge. Korean J. Plant Res..

[28] Lao X., Suwabe K., Niikura S., Kakita M., Iwano M., Takayama S. (2014). Physiological and genetic analysis of CO_2_-induced breakdown of self-incompatibility in *Brassica rapa*. J. Exp. Bot..

[29] Lv H., Miyaji N., Osabe K., Akter A., Mehraj H., Shea D.J., Fujimoto R., Kole C. (2020). The importance of genetic and epigenetic research in the Brassica vegetables in the face of climate change. Genomic Designing of Climate-Smart Vegetable Crops.

[30] Muñoz-Sanz J.V., Zuriaga E., Cruz-García F., McClure B., Romero C. (2020). Self-(In) compatibility systems: Target traits for crop-production, plant breeding, and biotechnology. Front. Plant Sci..

[31] Nasrallah J.B., Kao T.H., Goldberg M.L., Nasrallah M.E. (1985). A cDNA encoding an S-locus specific glycoprotein from *Brassica oleracea*. Nature.

[32] Takasaki T., Hatakeyama K., Suzuki G., Watanabe M., Isogai A., Hinata K. (2000). The S receptor kinase determines self-incompatibility in *brassica* stigma. Nature.

[33] Chen F., Yang Y., Li B., Liu Z., Khan F., Zhang T., Zhou G., Tu J., Shen J., Yi B. (2019). Functional analysis of M-locus protein kinase revealed a novel regulatory mechanism of self-incompatibility in *Brassica napus* L. Int. J. Mol. Sci..

[34] Azibi T., Hadj-Arab H., Lodé M., de Carvalho J.F., Trotoux G., Nègre S., Gilet M.M., Boutte J., Lucas J., Vekemans X. (2020). Impact of whole genome triplication on the evolutionary history and the functional dynamics of regulatory genes involved in *Brassica* self-incompatibility signalling pathway. Plant Reprod..

[35] Dou S., Zhang T., Tu J., Shen J., Yi B., Wen J., Fu T., Dai C., Ma C. (2021). Generation of novel self-incompatible *Brassica napus* by CRISPR/Cas9. Plant Biotechnol. J..

[36] Tung H.L., Alemany S., Cohen P. (1985). The protein phosphatases involved in cellular regulation: 2. Purification, subunit structure and properties of protein phosphatases-2Ao, 2A1, and 2A2 from rabbit skeletal muscle. Eur. J. Biochem..

[37] Seshacharyulu P., Pandey P., Datta K., Batra S.K. (2013). Phosphatase: PP2A structural importance, regulation and its aberrant expression in cancer. Cancer Lett..

[38] Cohen P. (1989). The structure and regulation of protein phosphatases. Annu. Rev. Biochem..

[39] Mayer-Jaekel R.E., Hemmings B.A. (1994). Protein phosphatase 2A−a ‘menage a trois’. Trends Cell Biol..

[40] Smith R.D., Walker J.C. (1996). Plant protein phosphatases. Annu. Rev. Plant Biol..

[41] Luan S. (2003). Protein phosphatases in plants. Annu. Rev. Plant Biol..

[42] MacKintosh C., Cohen P. (1989). Identification of high levels of type 1 and type 2A protein phosphatases in high levels of type 1 and type 2A protein phosphatases in higher plants. Biochem. J..

[43] Jagiello I., Donella-Deana A., Szczegielnia J., Pinna L.A., Muszyńska G. (1992). Identification of protein phosphatase activities in maize seedlings. Biochim. Biophys. Acta—Mol. Cell Res..

[44] Bialojan C., Takai A. (1988). Inhibitory effect of a marine-sponge toxin, okadaic acid, on protein phosphatases. Biochem. J..

[45] Scutt C.P., Fordham-Skelton A.P., Croy R.R.D. (1993). Okadaic acid causes breakdown of self-incompatibility in *Brassica oleracea*: Evidence for the involvement of protein phosphatases in the incompatible response. Sex. Plant Reprod..

[46] Lee G.H., Shin N.R., Park Y.D. (2020). Reverse genetics analysis of the 55-kDa B regulatory subunit of 2A serine/threonine protein phosphatase (PP2A) related to self-incompatibility in Chinese cabbage. Hortic. Sci. Technol..

[47] Yu S.G., Cho N.H., Kim J.H., Oh T.R., Kim W.T. (2021). Suppression of DRR1 results in the accumulation of insoluble ubiquitinated proteins, which impairs drought stress tolerance. J. Integr. Plant Biol..

[48] Son G.H., Moon J., Shelake R.M., Vuong U.T., Ingle R.A., Gassmann W., Kim J.Y., Kim S.H. (2021). Conserved Opposite Functions in Plant Resistance to Biotrophic and Necrotrophic Pathogens of the Immune Regulator SRFR1. Int. J. Mol. Sci..

[49] Wu M., Liu H., Lin Y., Chen J., Fu Y., Lou J., Wang F. (2020). In-frame and frame-shift editing of the EHd1 gene to develop ja-ponica rice with prolonged basic vegetative growth periods. Front. Plant Sci..

[50] Fan D., Liu T., Li C., Jiao B., Li S., Hou Y., Luo K. (2015). Efficient CRISPR/Cas9-mediated targeted mutagenesis in Populus in the first generation. Sci. Rep..

[51] Enciso-Rodriguez F., Manrique-Carpintero N.C., Nadakuduti S.S., Buell C.R., Zarka D., Douches D. (2019). Overcoming self-incompatibility in diploid potato using CRISPR-Cas9. Front. Plant Sci..

[52] Xu Z.S., Feng K., Xiong A.S. (2019). CRISPR/Cas9-mediated multiply targeted mutagenesis in orange and purple carrot plants. Mol. Biotechnol..

[53] Doll N.M., Gilles L.M., Gérentes M.F., Richard C., Just J., Fierlej Y., Borrelli V.M.G., Gendrot G., Ingram G.C., Rogowsky P.M. (2019). Single and multiple gene knockouts by CRISPR–Cas9 in maize. Plant Cell Rep..

[54] Ren C., Liu Y., Guo Y., Duan W., Fan P., Li S., Liang Z. (2021). Optimizing the CRISPR/Cas9 system for genome editing in grape by using grape promoters. Hortic. Res..

[55] Ma C., Liu M., Li Q., Si J., Ren X., Song H. (2019). Efficient BoPDS gene editing in cabbage by the CRISPR/Cas9 system. Hortic. Plant J..

[56] Lopez-obando M., Hoffmann B., Géry C., Guyon-Debast A., Téoulé E., Rameau C., Bonhomme S., Nogué F. (2016). Simple and efficient targeting of multiple genes through CRISPR-Cas9 in *physcomitrella* patens. G3 Genes Genomes Genet..

[57] Shan S., Soltis P.S., Soltis D.E., Yang B. (2020). Considerations in adapting CRISPR/Cas9 in nongenetic model plant systems. Appl. Plant Sci..

[58] Pramanik D., Shelake R.M., Park J., Kim M.J., Hwang I., Park Y., Kim J.Y. (2021). CRISPR/Cas9-mediated generation of pathogen-resistant tomato against tomato yellow leaf curl virus and powdery mildew. Int. J. Mol. Sci..

[59] Elorriaga E., Klocko A.L., Ma C., Strauss S.H. (2018). Variation in mutation spectra among CRISPR/Cas9 mutagenized poplars. Front. Plant Sci..

[60] Zhang J., Guo D., Chang Y., You C., Li X., Dai X., Weng Q., Zhang J., Chen G., Li X. (2007). Non-random distribution of T-DNA insertions at various levels of the genome hierarchy as revealed by analyzing 13 804 T-DNA flanking sequences from an enhancer-trap mutant library. Plant J..

[61] Pla M. (2012). Insert stability and transgenic plant risk. Encyclopedia of Biotechnology in Agriculture and Food.

[62] Forsbach B., Schubert D., Lechtenberg B., Gils M., Schmidt R. (2003). A comprehensive characterization of single copy T-DNA insertions in the *Arabidopsis thaliana* genome. Plant Mol. Biol..

[63] Peterson B.A., Haak D.C., Nishimura M.T., Teixeira P.J.P.L., James S.R., Dangl J.L., Nimchuk Z.L. (2016). Genome-wide assessment of efficiency and specificity in CRISPR/Cas9 mediated multiple site targeting in Arabidopsis. PLoS ONE.

[64] Hahn F., Nekrasov V. (2018). CRISPR/Cas precision: Do we need to worry about off-targeting in plants?. Plant Cell Rep..

[65] Tripathi L., Ntui V.O., Tripathi J.N. (2021). RNA interference and CRISPR/Cas9 applications for virus resistance. CRISPR and RNAi Systems: Nanobiotechnology Approaches to Plant Breeding and Protection.

[66] Nishihara M., Higuchi A., Watanabe A., Tasaki K. (2018). Application of the CRISPR/Cas9 system for modification of flower color in *Torenia fournieri*. BMC Plant Biol..

[67] Charrier A., Vergne E., Dousset N., Richer A., Petiteau A., Chevreau E. (2019). Efficient targeted mutagenesis in apple and first time edition of pear using the CRISPR-Cas9 system. Front. Plant Sci..

[68] Zhou E., Zhang Y., Wang H., Jia Z., Wang X., Wen J., Shen J., Fu T., Yi B. (2022). Identification and Characterization of the MIKC-Type MADS-Box Gene Family in *Brassica napus* and Its Role in Floral Transition. Int. J. Mol. Sci..

[69] Martínez M.I.S., Bracuto V., Koseoglou E., Appiano M., Jacobsen E., Visser R.G., Wolters A.M.A., Bai Y. (2020). CRISPR/Cas9-targeted mutagenesis of the tomato susceptibility gene *PMR4* for resistance against powdery mildew. BMC Plant Biol..

[70] Navet N., Tian M.Y. (2020). Efficient targeted mutagenesis in allotetraploid sweet basil by CRISPR/Cas9. Plant Direct.

[71] Wan D.Y., Guo Y., Cheng Y., Hu Y., Xiao S., Wang Y., Wen Y.Q. (2020). CRISPR/Cas9-mediated mutagenesis of *VvMLO3* results in enhanced resistance to powdery mildew in grapevine (*Vitis vinifera*). Hort. Res..

[72] Kim H.R., Kim S.T., Ryu J.H., Choi M.K., Kweon J.Y., Kang B.C., Ahn H.M., Bae S.J., Kim J.G., Kim J.S. (2016). A simple, flexible and high-throughput cloning system for plant genome editing via CRISPR-Cas system. J. Integr. Plant Biol..

[73] Jyothishwaran G., Kotresha D., Selvaraj T., Srideshikan S.H., Rajvanshi P.K., Jayabaskaran C. (2007). A modified freeze-thaw method for efficient transformation of *Agrobacterium tumefaciens*. Curr. Sci..

[74] Murashige T., Skoog F. (1962). A revised medium for rapid growth and bio assays with tobacco tissue cultures. Physiol. Plant.

[75] Lee M.K., Kim H.S., Kim J.S., Kim S.H., Park Y.D. (2004). Agrobacterium-mediated transformation system for large-scale producion of transgenic Chinese cabbage (*Brassica rapa* L. ssp. pekinensis) plants for insertional mutagenesis. J. Plant Biol..

[76] McCouch S.R., Kochert G., Yu Z.H., Wang Z.Y., Khush G.S., Coffman W.R., Tanksley S.D. (1988). Molecular mapping of rice chromosomes. Theor. Appl. Genet..

