# Supplementary material for: Function Analysis of the PR55/B Gene Related to Self-Incompatibility in Chinese Cabbage Using CRISPR/Cas9
Source: Int J Mol Sci. 2022 May 3;23(9):5062. doi: 10.3390/ijms23095062 (PMC9102814; doi:10.3390/ijms23095062)
Supplement: Supplementary file 1 [file ijms-23-05062-s001.zip › ijms-1671490-supplementary.pdf]

Supplementary materials

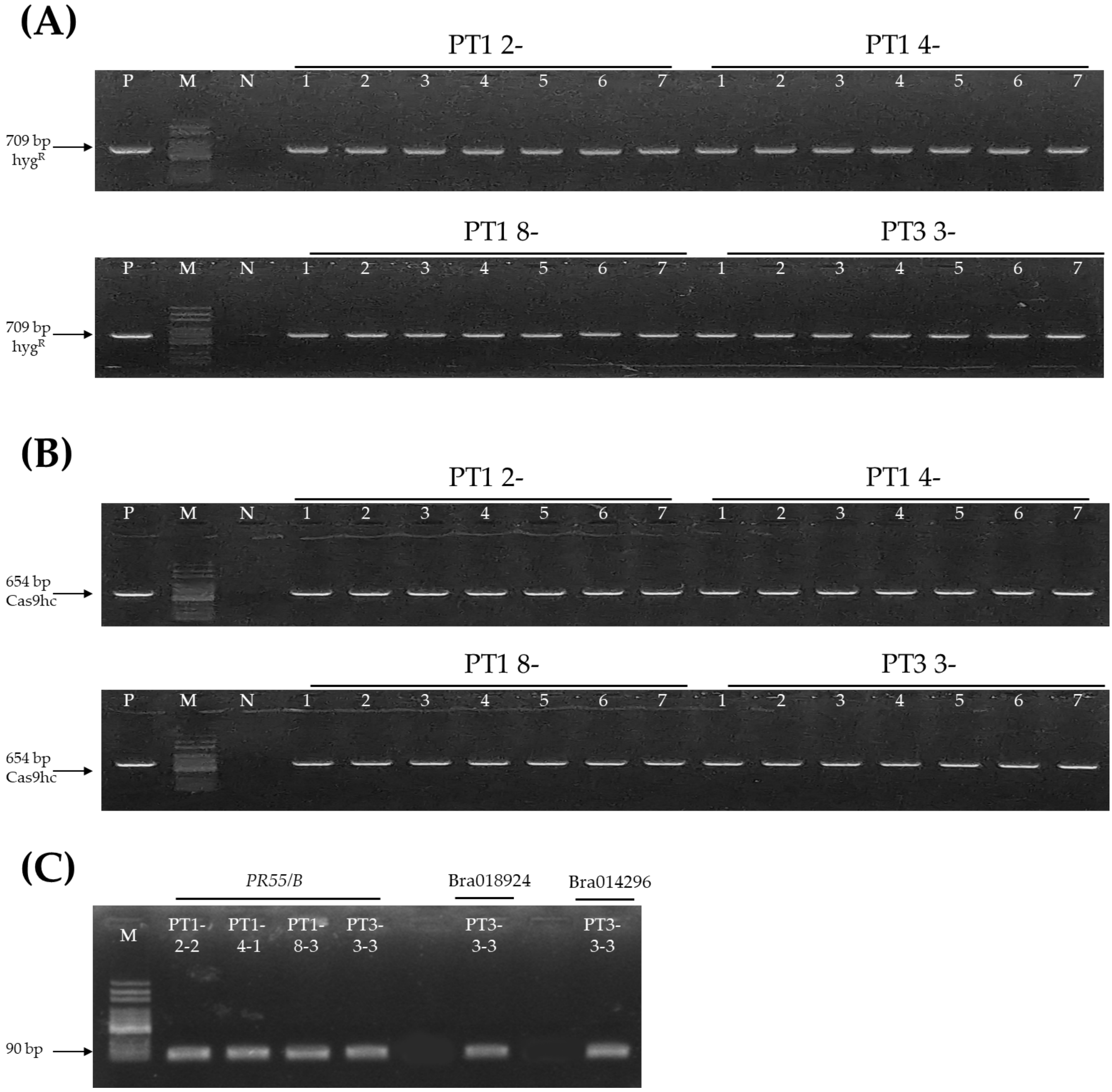

Figure S1. Selection of T_1 gene-edited lines by PCR and RT-PCR analysis. (**A**) PCR analysis with hyg^R primer sets of T_1 gene-edited lines. (**B**) PCR analysis with Cas9hc primer sets of T_1 gene-edited lines. The 709 bp and 654 bp expected PCR products are indicated with an arrow, respectively. P, positive control; M, 100 bp DNA ladder; N, negative control; Numbering lane, gene-edited lines. (**C**) RT-PCR analysis with gene-specific primer sets of T_1 gene-edited lines. The 90bp expected RT-PCR products were amplified. P, positive control; M, 100 bp DNA ladder; N, negative control; Numbering lane, gene-edited lines.

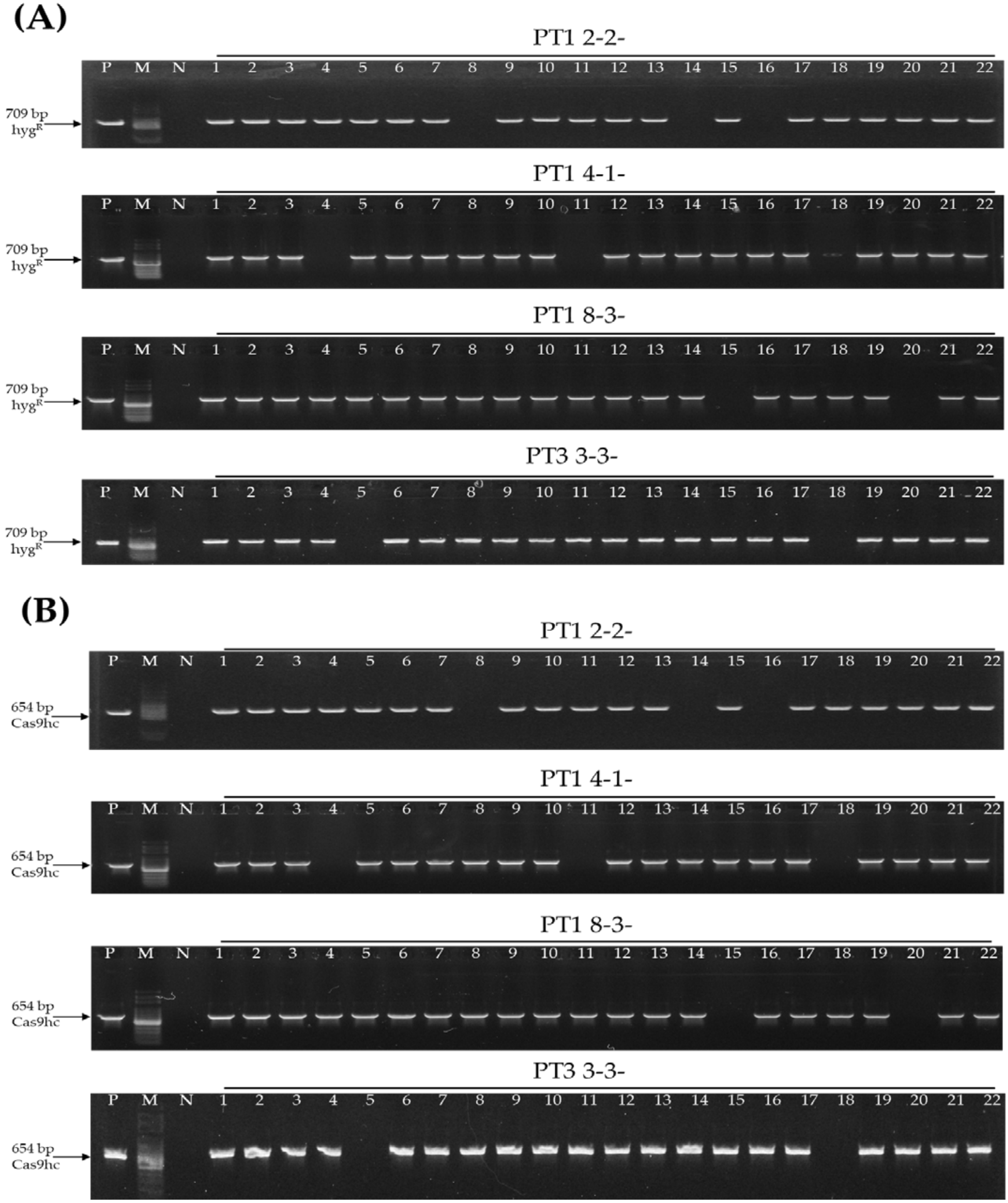

Figure S2. Selection of T_2 gene-edited lines by PCR analysis. (**A**) PCR analysis with hyg^R primer sets of T_2 gene-edited lines. (**B**) PCR analysis with Cas9hc primer sets of T_2 gene-edited lines. The 709 bp and 654 bp expected PCR products are indicated with an arrow, respectively. P, positive control; M, 100 bp DNA ladder; N, negative control; Numbering lane, gene-edited lines.

Table S1. List of primer sets for PCR and RT-PCR analysis.

Name	Primer	Sequence (5'→3')	Expected product size (bp)
**hyg^R**	F[z]	CGT CTG CTG CTC CAT ACA AG	709
	R	TGT CGA GAA GTT TCT GAT CGA	
**Cas9hc**	F	CCG CCA GGA GGA CTT CTA CC	654
	R	ATG TTC TCG GGC TTG TGG CG	
**PT1 cSEQ030425**	F	CTC AGG GTC ATG ACT CTG AAA	80
	R	GGG ATC TTG AAG ACA CTG ATC A	
**PT3 cSEQ030425**	F	GCA GCA GGT CCT AAG TCG TT	91
	R	ACG GCT AAG GAG ATA TCT TCC	
**PT3 cSEQ018924**	F	TTG AGG AAC CAG ATG CA	103
	R	CGG CTA AGT AAG TAT CTT CCT	
**PT3 cSEQ014296**	F	CAC TGA GAT TAT TGC TTC AG	85
	R	CTT AAG TGT CAT GTA GTC ACG	
	R	GCA GCA GGT CCT AAG TCG TT	

[Z]F, forward primer; R, reverse primer.
